# Return to Work and Quality of Life after Stroke in Italy: A Study on the Efficacy of Technologically Assisted Neurorehabilitation

**DOI:** 10.3390/ijerph17145233

**Published:** 2020-07-20

**Authors:** Sheyda Ghanbari Ghoshchi, Sara De Angelis, Giovanni Morone, Monica Panigazzi, Benedetta Persechino, Marco Tramontano, Edda Capodaglio, Pierluigi Zoccolotti, Stefano Paolucci, Marco Iosa

**Affiliations:** 1 IRCCS Fondazione Santa Lucia, Via Ardeatina 306, 00179 Rome, Italy; sheyda.ghanbarighoshchi@uniroma1.it (S.G.G.); s.deangelis@hsantalucia.it (S.D.A.); g.morone@hsantalucia.it (G.M.); m.tramontano@hsantalucia.it (M.T.); pierluigi.zoccolotti@uniroma1.it (P.Z.); s.paolucci@hsantalucia.it (S.P.); 2 Department of Psychology, Sapienza University of Rome, 00185 Rome, Italy; 3 Istituti Clinici Scientifici Maugeri IRCSS, Occupational Therapy and Ergonomics Unit, 27040 Montescano, Italy; monica.panigazzi@icsmaugeri.it; 4 Istituti Clinici Scientifici Maugeri IRCSS, Occupational Therapy and Ergonomics Unit, 27100 Pavia, Italy; edda.capodaglio@icsmaugeri.it; 5 Italian Workers’ Compensation Authority (INAIL), Department of Occupational and Environmental Medicine, Epidemiology and Hygiene, Monte Porzio Catone, 00078 Rome, Italy; b.persechino@inail.it; 6 Department of Movement, Human and Health Sciences, University of Rome “Foro Italico”, Interuniversity Centre of Bioengineering of the Human Neuromusculoskeletal System, 00135 Rome, Italy

**Keywords:** stroke, wearable devices, return to work, rehabilitation, kinematics, quality of life

## Abstract

Cerebrovascular diseases, including stroke, are historically considered diseases of old adults so only in a few studies has “return to work” (RTW) been considered as an index of rehabilitative outcome. At the moment, data on RTW in patients with stroke are highly variable: four different reviews reported the following ranges: 11–85%, 19–73%, 22–53%, and 40–45%. The absence of re-integration to work after a stroke is shown to be associated with an increase of cardiac disorders and depression, with a higher level of mortality, with social isolation and with insufficient adaptive skills. The aim of this study was to verify the effectiveness of technological treatment, performed with optic (SonicHand) and wearable (Riablo™) systems providing auditory and visual biofeedback, on RTW in patients with stroke. RTW was found to be associated with a higher independence in the activities of daily living (assessed by the Modified Barthel Index). No significant differences were found between technological versus conventional rehabilitation in terms of RTW, despite the former showing a higher odds ratio than the latter (OR = 9 vs. 6). Assistive devices were mainly used in patients who had not returned to work. Finally, quality of life was found higher in those patients who returned to work with the same conditions (work duties and time) as before stroke.

## 1. Introduction

Stroke is often considered a disease related to the elderly. In most studies, the rehabilitation outcome is the return to a proper level of independence in daily living activities. Few studies have used “return to work” (RTW) as an outcome index. 

In the meantime, the mean age of stroke onset has decreased, with an increment of the incidence of stroke under 55 [1], and the retirement age in Italy has increased. The result is that the incidence of stroke in the working-age population has increased, and a higher number of people may be led to consider RTW as an important end-point of recovery after neurorehabilitation. The absence of re-integration at work after a stroke is shown to be associated with an increase of depression, social isolation, insufficient adaptive skills, and even with cardiac disorders and a higher level of mortality [2,3]. 

In Italy, the object of the present study, data on RTW and the recovery of social participation showed that only 19% of people after a stroke get back to work [4].

Return to work can be considered the highest point of the reintegration process, with positive consequences for the patient’s health and well-being [5,6]. However, this stage is also very delicate, requiring individualized compliance with work activity in its organizational, structural and ergonomic aspects for this complex type of patient [5].

Hence, there is the need for greater accuracy in therapeutic choices and in controlling and rationalizing health care costs, and greater efficiency in achieving results optimized for return to work and quality of life, which requires a complex and very difficult approach [7]. 

Developments in technology-assisted rehabilitation have made possible the formulation of new rehabilitation paradigms derived from the integration of conventional rehabilitative programs with instrumental interventions specifically selected for the recovery of specific functions completely or partially lost with stroke [8,9]. These technologies may also allow professionals to have continuous and accurate data monitoring along with a summary of trends, that important for understanding and testing the effectiveness of treatments and the impact of observed variables [10,11]. 

In fact, the rehabilitation project should also aim at recovering functional capacity as well as motor skills and social reintegration, along with RTW, key objectives in recovery after a disabling pathology such as stroke.

These new technologies for rehabilitation (i.e., instrumented biofeedback, serious exergames, virtual reality, collaborative robots [12]) may help patients with stroke to obtain a complete or, at least, a satisfying recovery of gross and fine motor functions, and also may favor individual autonomy regarding everyday life activities, including return to work.

Among these technologies, miniaturized and wireless wearable or optical sensors are increasingly being used for quantitative instrumental-based assessment of patients’ functions and for their rehabilitation in neurological disorders [13,14,15,16]. 

Among these technologies, the sonification of hand and upper limb movements, using an optoelectronic device as biofeedback, has been recently proposed [17,18], as have exergames for videogame-based therapy, controlled by wearable devices and a balance platform for the rehabilitation of postural and limb control and balance rehabilitation [19]. These technologically-assisted interventions may enhance the compliance of patients and, most importantly, act on neuronal mechanisms for re-learning through positive reinforcement [20]. 

The aim of this study is to assess return to work and the quality of life of patients with stroke after conventional rehabilitation and technological rehabilitation (performed with wearable and/or optic assistive devices), while identifying the prognostic factors for a satisfactory RTW.

## 2. Materials and Methods 

### 2.1. Trial Design

The study design conformed to CONSORT Guidelines for a two-arm, single-blind randomized-controlled trial. The trial was approved by the Local Ethics Committee. All participants gave their written informed consent for participation in the study. A researcher who was not involved in the intervention sessions assessed the patients’ eligibility to participate, based on the inclusion and exclusion criteria. 

### 2.2. Participants

This study was carried out in three Italian neurorehabilitation hospitals (IRCCS Fondazione Santa Lucia of Rome, IRCCS ICS Maugeri of Pavia and IRCCS ICS Maugeri of Montescano), where the enrolled patients had been hospitalized. In total, 50 patients with a diagnosis of stroke (> 6 months after stroke) were recruited according to the following inclusion criteria: subjects with a diagnosis of stroke occurring in working age (aged between 18 and 66 years) and with work activity at the time of the acute adverse event. Exclusion criteria included cognitive impairments within MMS (Mini-Mental State Examination) < 24, severe unilateral spatial neglect (assessed by the Letter Cancellation test, Barrage test, the Salford Sentence Reading Test, and the Wundt-Jastrow Area Illusion Test) and any severe comorbidities. Participants were randomly assigned to one of two groups; randomization was carried out by means of a computer-generated random numbers system. The two groups were the Technological Rehabilitation group (TG) and Control conventional rehabilitation Group (CG), as detailed in the following.

### 2.3. Interventions

All patients performed rehabilitation in the day-hospital regimen. Both groups performed the same number of rehabilitation sessions: two sessions of neuromotor rehabilitation per day and one session either of speech therapy, respiratory or phoniatric rehabilitation, three days per week for one month, each session lasting 40 min. Both groups received the same amount of therapy. For TG, as detailed below, four hundred minutes of the above reported sessions of neuromotor rehabilitation were performed using technological devices. The CG performed all the above rehabilitation sessions according to conventional therapy, based on customized exercises focused either on hand dexterity and hand fine motor rehabilitation or on balance and postural improvement, according to prescribed physical exercises and rehabilitation targets, including the use of stabilization techniques and target achievements for the recovery of independence in the activities of daily living.

As stated above, patients allocated to the TG performed the same amount of therapy as the CG, but with the difference that four hundred minutes of the total hours of motor neurorehabilitation were performed using the SonicHand [21] or the Riablo ™ [19], according to the individual established rehabilitation objectives defined by the clinical staff in accordance with the patient’s expectancies. Both these devices provided technological biofeedback related to patients’ movements. Riablo treatment was administered to patients with deficits related to postural balance and limb gross motor functions, but able to maintain upright posture at least for the time needed to perform the required tasks for the device [19]. SonicHand treatment was administered to patients with hand deficits and needing rehabilitation of upper limb fine motor functions and hand dexterity [17,21]. Despite some patients having both types of deficit, they received only one type from these two technologically assisted therapies. In these cases, two allocation criteria were followed: if the patients were confined toa wheelchair they were allocated to the SonicHand subgroup (because Riablo treatment cannot be administered to them); if patients were able to maintain the upright posture they received treatment according to personalized neurorehabilitation taking into account the most severe deficits (hand deficits vs. gross motor deficits) and the expectancies of patients and their families. The first of these two criteria led to SonicHand treatment being allocated to some of the most severely affected patients.

The SonicHand rehabilitation protocol [17] was in two phases. The first phase consisted of a 10 minutes warm-up including the exercises, complying with the individual needs and motor rehabilitation of each patient and chosen from wrist mobilization, opening and closing fingers and pronation and supination of the forearm. In the second phase which lasted for 10 minutes, at least six different exercises were proposed for the patient, choosing from: wrist radial and ulnar deviation, flexion and extension, pronation and supination; hand grasping, pinching and extension; shoulder and elbow flexion and extension and forward thrust. During these exercises, the movements were supported by the “sonification” technique. The sonification process was made using the Leap Motion Controller (LMC^®^; Leap Motion, Inc., San Francisco, CA, USA), an optic assistive device, the software of which was adapted for rehabilitative purposes. The LMC^®^ is a human computer interface able to capture hand and finger movements and these data were transformed in sounds, thanks to a custom application developed with the Max 7 software programming environment (LMC^®^; Leap Motion, Inc., San Francisco, CA, USA). The software program generated music sequences according to hand movements detected by the LMC^®^. The application is able to associate movement with a four-note arpeggio or with a modulated texture. Based on the chosen exercises, sound parameters such as pitch, volume and spectrum have been identified and associated with the movement. The correct execution of the required movement allowed the production of a harmonic progression of arpeggios. The Sonic Hand protocol was performed for 20 sessions each lasting for 20 minutes. 

The Riablo™ (CoRehab, Trento, Italy) is an adaptive system consisting of several wearable sensors and a stabilemetric platform wirelessly connected to software, to provide biofeedback through a video interface [19]. It allows the patients to enhance standard rehabilitation of balance and gross motor functions by guiding user’s performances through the use of video-game based therapy. A protocol with six different exercises was proposed: the latero-lateral load shift; displacement of latero-lateral load on an oscillating platform; antero-posterior load shift; displacement of antero-posterior load on the oscillating platform; displacement of latero-lateral load with knee flexion; and lateral load displacement with knee flexion on an oscillating platform. All subjects performed 10 Riablo™ training sessions, each lasting 40 minutes with an initial preparatory phase. 

### 2.4. Outcomes

All patients were assessed with the Modified Barthel Index (MBI) [22], both at admission time (T0) and at discharge (T1). After almost six months after discharge, patients were contacted for a post-discharge telephone follow-up (FU). 

During the phone call, all patients were assessed using the MBI [22,23] and the 12-Item Short Form Health Survey (SF-12) [24,25]. A specific questionnaire for RTW was also administered, together with questions related to any organizational or physical adaptations applied for the workplace. The Quebec User Evaluation of Satisfaction with Assistive Technology (QUEST) [26] and the Individual Prioritized Problems Assessment (IPPA) [27] were also administered but only to patients who used a specific assistive devices, prescribed by a physiatrist to patients during rehabilitation after stroke for increasing their independence in activities of daily living. These aids consisted of tripods, poles, wheelchairs or walkers. These were provided independently of belonging to the CG or TG and they did not refer to SonicHand or Riablo, but were provided to patients on the basis of physician assessment and of the detection of particular individual needs in order to cope with the autonomous execution of functional activities.

All the evaluation scores and the questionnaire data were collected by a researcher blinded to the allocation group.

### 2.5. Statistical Analysis

Binary data was reported using percentage values and analysed by χ^2^-test. Ordinal scores were reported using median and first and third quartiles and analysed using Mann-Whitney u-test. Continuous measures were reported using mean ± standard deviations and analysed using a t-test. Forward binary logistic regression was used for identifying prognostic factors, and non-binary parameters were dichotomized using their median value. This last analysis allowed for computing the odds ratios (OR) and the relevant 95% confidence intervals (CI95%). For all the analyses the alpha level of statistical significance was set at 5%.

## 3. Results

**Figure 1:**
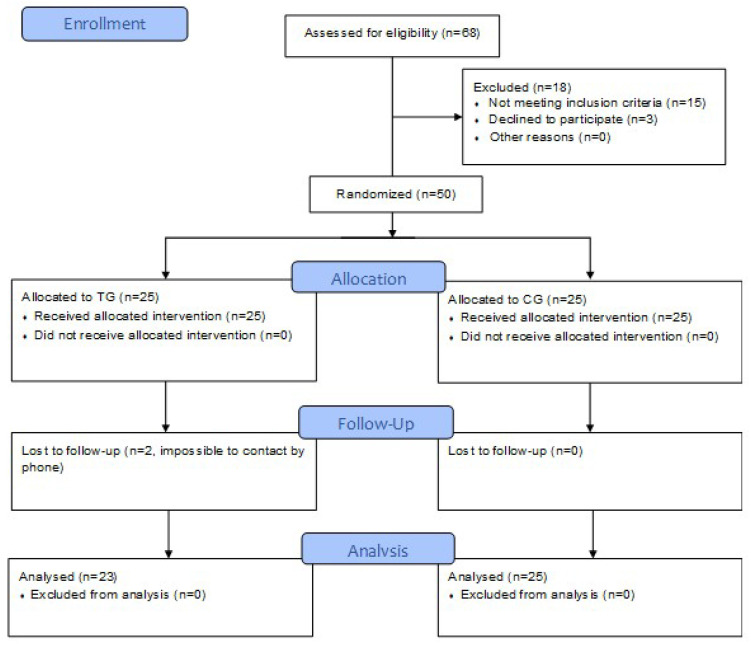
Flow Diagram.

The final analysed sample consisted of 48 patients with stroke (two patients of TG were not traceable at follow-up), of whom 20 had returned to work (Figure 1). Data of the samples are reported in Table 1 for patients enrolled in TG and those in CG. The two groups were roughly balanced at admission, and no significant differences were found between them at further assessment. Table 1 also shows a comparison within TG of patients treated with Riablo (*N* = 10) and those with SonicHand (*N* = 13). Significant differences were found between these two subgroups for MBI-scores at the three assessment times and in the number of patients who experienced at least one fall after discharge, but not in terms of likelihood to return to work or quality of life. 

**Figure 2:**
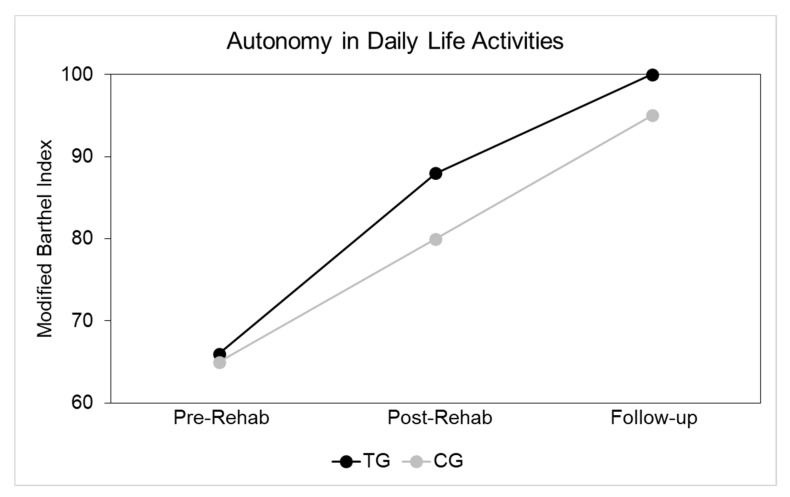
Median MBI-scores at admission, discharge and follow-up for control group (CG) (grey) and technological rehabilitation group (TG) (black).

Seeing as TG and CG were not significantly different, the data could be combined for the entire sample, as done in Table 2, in which comparisons were further performed between patients who returned to work and those who did not. Statistically significant differences were found between these patients in terms of MBI-scores at follow-up, physical score on SF-12 and number of patients needing an aid. At follow-up, 13 patients were using an assistive device. Table 3 reports features of patients who returned to work obtained by a specific questionnaire. Again, no significant differences were obtained between TG and CG patients. The MBI-scores at admission, discharge and follow-up are shown in Figure 2. No statistically significant differences were recorded between the two groups at each evaluation time. 

Table 4 shows the results of the two Forward Binary Logistic Regressions. The first regression was performed using return to work as the main outcome, and hence as a dependent variable. The only factor found to be statistically influencing RTW was the MBI score at follow-up, with a probability of 7.5 times higher for patients who had an MBI equal or higher than 95 to return to work compared to other patients. A further analysis was conducted, separating TG and CG on the possibilities of returning to work, showing an Odds Ratio OR = 9 (*p* = 0.027) and OR = 6 (*p* = 0.05), respectively. The second regression analysis showed that a good quality of life, assessed by an SF-12 total score higher than 95, was influenced by RTW on the same conditions as before stroke (OR = 6.6, *p* = 0.026), and neither just by RTW (*p* = 0.613), nor by a good level of autonomy (MBI f-up: *p* = 0.383). Eleven subjects returned to work on the same conditions, same work duties and same work times as before the stroke event. 

## 4. Discussion

Participation in the work role is important as it enhances social reintegration and may influence quality of life, but the percentage of patients returning to work after stroke is still low. In this study, RTW occurred in 42% of subjects. These results are in line with previously reported data of 40–45% RTW [4,28,29]. Other studies have reported higher variability for RTW: 11–85% [28], 19–73% [4], or 22–53% [29]. In our specific study, the features of patients returned to work are those reported in Table 3: they were more independent in the activities of daily living (MBI at follow-up), with a higher quality of life in the physical domain, and fewer needed an assistive device when compared to those not returned to work. These results were summarized by logistic regression analysis in the identification by Modified Barthel Index assessed at follow-up as the only consistent prognostic factor related to RTW. In our study, age did not show a statistically significant relation to RTW. However, after a stroke, one of the main determinants of returning to work seems to be working age [28,30,31,32]. Barker-Collo et al. [33] showed that age was an important predictor of RTW. The discrepancy with our results may be due to the fact that age may have indirectly influenced RTW by affecting the level of independence in the activities of daily living, a factor directly assessed in our study by means of MBI-fup. Despite this parameter being slightly higher in the technologically treated group, the effect of this type of treatment was not significantly higher than that of conventional treatment. These results did not support previous studies reporting that new modern technological interventions have a higher positive impact on stroke rehabilitation than conventional interventions [34,35]. Patients allocated to the technological group could be treated with SonicHand if their main deficit was related to the hand, or with Riablo if they had postural and limb gross motor deficits. Severe patients confined to wheelchair could perform the SonicHand treatment, but not Riablo, because this last device needs an upright posture for performing balance and limb motor training. It was expected that the technological subgroup treated with SonicHand would be more problematic than that treated with Riablo, showing significantly lower scores of MBI in all three assessment times. The different severity and also the specific effects of Riablo treatment, on balance [19], may explain the reasons for which none of the patients treated with Riablo experienced a fall after discharge, whereas 38% of patients treated with SonicHand (and 25% of CG) fell at least once after discharge. Despite these differences, neither likelihood of return to work nor quality of life was significantly different between the two technological subgroups.

We found only an indirect effect of technological rehabilitation: a significant higher odds ratio was found in favor of technological rehabilitation versus conventional in achieving a higher MBI-score at follow-up; this latter parameter was then found to be directly influencing RTW. It seems that, with the same autonomy achieved, patients treated with technological rehabilitation are more likely to return to work than those treated with conventional rehabilitation. At the same time, conventional therapy has shown the advantage of also allowing some less independent patients to return to work. These findings are in line with previous studies showing that technological rehabilitation could be more effective for specific patients (depending on the degree of severity with respect to the type of technology), whereas conventional therapy could have a generalized positive effect [36,37,38].

One of the most interesting results of our study was that return to work by itself was not sufficient to warrant good quality of life. Quality of life was higher in subjects who returned to work without any losses in terms of time or working duties. To achieve this, a highly specialized rehabilitation is needed, and this should be integrated with specific interventions in the workplace. Conversely, in our study, only one patient of those who returned to work received an assistive device, showing that there is the need to improve the culture of RTW after stroke, satisfying the needs of subjects for a complete recovery of job status similar to that enjoyed before the stroke. In fact, only subjects who returned to the same job and at the same working conditions as before stroke reported a high quality of life.

Our study has some limitations. The dimension of the workplace and specific characteristics of the job were not investigated, as well as social characteristics of the job. Despite the fact that we recorded this information during phone follow-up, the sample size of our study limited the significance of the comparisons between the different technologies used in TG and did not allow us to divide subjects in subgroups related to the type of their job. The economic impact on the family of patients relating to RTW was not assessed in this study, but this could be a fundamental aspect leading the decision not to return to work in patients with moderate independence in the activities of daily living. Finally, psychological aspects should be investigated in further studies to analyze the factors influencing RTW and those influenced by RTW, together with other possible factors, such as pain [39] and patients’ needs [40]. Along these lines, psychosocial support should be considered as a critical aspect of stroke rehabilitation. Technological rehabilitation could be helpful for people who use technological devices in their work but may be less useful in subjects not used to interacting with modern technologies [41,42,43].

In conclusion, our study has shown that quality of life after stroke is strongly influenced by the possibility of returning to work under the same conditions as before the acute event. This may depend on the independence achieved by patients, which was not superior under one type of rehabilitation versus the other type. Furthermore, patients enrolled in our study achieved a good level of independence, highlighting the importance of personalized rehabilitative treatments focused on the characteristics and the needs of each patient. Finally, our study recommends institutions to favor the development of facilities in workplaces that can accommodate patients and help them to adapt to their job, without the constraint of reduced working duties or working time.

## Figures and Tables

**Table 1:** Patients (P.) data at the follow-up: absolute frequency (and percentages) for discrete variables, mean ± standard deviation for continuous variables, median (and first and third quartiles) for ordinal data. The *p*-values (in bold if statistically significant) refer to the comparison between patients enrolled in technological group (TG) and those enrolled in the conventional therapy group (CG) in the fourth column and between patients who received Riablo versus SonicHand treatments in the sixth column. These p-values were obtained by the tests reported in the last column and selected according to the variable types.

Demographical and Clinical Data	TG (*N* = 23)	CG (*N* = 25)	*p*-Value	Riablo (*N* = 10)	SonicHand (*N* = 13)	*p*-Value	Test
Age (years)	51.0 ± 11.8	52.5 ± 10.5	0.648	47.5 ± 9.1	54.0 ± 13.3	0.221	t-test
Months from stroke	27.0 ± 13.2	21.7 ± 12.2	0.156	26.6 ± 6.5	27.0 ± 16.9	0.902	t-test
Male sex	14 (60.9%)	17 (68.0%)	0.606	7 (70%)	7 (53.8%)	0.431	χ^2^-test
MBI Pre-Rehab	66 (41;78)	65 (19; 75)	0.793	79 (74; 82)	45 (25; 60)	**0.002**	u-test
MBI Post-Rehab	88 (72;98)	80 (65; 90)	0.391	98 (95; 100)	80 (65; 87)	**0.005**	u-test
MBI follow-up	100 (82;100)	95 (85;100)	0.450	100 (100; 100)	90 (65; 100)	**0.016**	u-test
SF-12 total	94 (83;106)	97 (84; 104)	0.543	104 (87; 109)	92 (75; 109)	0.343	u-test
SF-12 physical	47 (39;54)	43 (36; 49)	0.190	48 (45; 53)	43 (31; 54)	0.563	u-test
SF-12 mental	51 (42;58)	58 (48; 59)	0.599	55 (47; 59)	50 (38; 56)	0.284	u-test
P. with assistive devices	5 (21.7%)	8 (32.0%)	0.424	2 (20%)	3 (23.1%)	0.859	χ^2^-test
QUEST 1 (*N* = 13)	4.7 (4.4;5.0)	4.8 (4; 4.8)	0.602	4.7 (4.6; 4.9)	4.7 (4.6; 4.9)	0.767	u-test
QUEST 2 (*N* = 13)	5 (0.0;5.0)	2.5 (0; 4.6)	0.194	2.4 (1.2; 3.6)	5 (5; 5)	0.053	u-test
IPPA 1 (*N* = 13)	20 (19;23.3)	23 (18.4;25.0)	0.303	19.5 (19.2;19.7)	20 (15; 21.6)	0.767	u-test
IPPA 2 (*N* = 13)	10 (8;10.5)	15 (10.5; 20)	0.107	11.5 (11.2;11.7)	7 (6.8; 8.5)	0.083	u-test
IPPA Difference (*N* = 13)	8.0 (5.0;10)	7.8 (5; 10.2)	0.605	8 (8; 8)	10 (6.5;13.3)	0.554	u-test
P. with fall events	5 (21.7%)	4 (16.0%)	0.611	0 (0%)	5 (38.5%)	**0.026**	χ^2^-test
Return to Work	11 (47.8%)	9 (36.0%)	0.406	7 (70%)	4 (30.8%)	0.062	χ^2^-test

**Table 2:** Patient data at follow-up: absolute frequency (and percentages) for discrete variables, mean ± standard deviation for continuous variables, median (and first and third quartiles) for ordinal data. The *p*-values (in bold if statistically significant) refer to the comparison between subjects returned and not returned to work computed using the test reported in the last column according to the variable types.

Demographical and Clinical Data	Entire Sample	Returned to Work	Not Returned to Work	*p*-Value	Test
Number of subjects	48	20 (42%)	28 (58%)	0.248	χ^2^-test
Age (years)	51.8 ± 11.1	53.9 ± 8.6	50.3 ± 12.5	0.264	t-test
Time from stroke (months)	24.2 ± 12.8	25.6 ± 12.0	23.2 ± 13.5	0.528	t-test
Male sex	31 (64.6%)	13 (65.0%)	18 (64.3%)	0.959	χ^2^-test
MBI follow-up	100 (91;100)	100 (100;100)	100 (70;100)	**0.018**	u-test
SF-12 total	96 (83;107)	100 (87;109)	93 (77; 105)	0.143	u-test
SF-12 physical	45 (36;51)	48 (44; 55)	43 (33; 48)	**0.009**	u-test
SF-12 mental	52 (42;59)	53 (41; 59)	52 (43; 58)	0.983	u-test
Patients with assistive devices	13 (27.1%)	1 (5.0%)	12 (42.8%)	**0.004**	χ^2^-test
QUEST 1 (N = 13)	4.7 (4.1;5.0)	4.8	4.7 (4.4;5)	0.683	u-test
QUEST 2 (N = 13)	4.5 (0.0;5.0)	0	4.6 (0.7;5)	0.209	u-test
IPPA 1 (N = 13)	20 (17.8;24.1)	25	20 (18.4;23.3)	0.227	u-test
IPPA 2 (N = 13)	11.3 (7.5;18.9)	20	11.2 (7.7;13.7)	0.228	u-test
IPPA Difference (N = 13)	8.0 (5.0;10.5)	5	8.0 (7.0;10.3)	0.346	u-test
Patients with fall events	9 (18.7%)	2 (10.0%)	7 (25.0%)	0.189	χ^2^-test
Technological treatment	23 (47.9%)	11 (55.0%)	12 (42.9%)	0.406	χ^2^-test

**Table 3:** Data of patients returned to work and comparison of TG and CG.

Return to Work Data	Entire Sample of Returned to Work	Technological Treatment Group	Conventional Treatment Group	*p*-Value	Test
Number of subjects	20	11	9	0.655	χ^2^-test
Working Hours	32.6 ± 9.3	33.7 ± 9.0	31.1 ± 10.1	0.552	χ^2^-test
People with any kind of adaptation	9 (45.0%)	4 (36.4%)	5 (55.5%)	0.888	χ^2^-test
People with job adaptation	4 (20.0%)	1 (9.1%)	3 (33.3%)	0.391	χ^2^-test
People with time adaptation	7 (35.0%)	4 (36.4%)	3 (33.3%)	0.178	χ^2^-test
People with tools adaptation	0 (0%)	0 (0%)	0 (0%)	-	-
People with assistive devices	1 (5.0%)	0 (0%)	1 (11.1%)	0.257	χ^2^-test

**Table 4:** Results of Forward Binary Logistic Regression (OR: odds ratio; CI95%: 95% confidence interval, p-Value in bold if statistically significant).

Dependent Variable	Variables into the Model	OR	*p*-Value	CI95%	Explained Variance	Variables out of the Model
Return to work	MBI f-up	7.5	**0.002**	2.04; 27.59	72.9%	Age: *p* = 0.527 Gender: *p* = 0.805 Treatment: *p* = 0.728 Time from stroke: *p* = 0.610 MBI pre-R: =0.128 MBI post-R = 0.740
SF-12 Total score	Work as before (job type and time)	6.6	**0.026**	1.2; 34.9	64.6%	Age: *p* = 0.459 Treatment: *p* = 0.449 Time from stroke: *p* = 0.174 Gender: *p* = 0.497 Return to work: *p* = 0.613 MBI pre-R: *p* = 0.331 MBI post-R: *p* = 0.269 MBI f-up: *p* = 0.383

## Data Availability

The data that support the findings of this study are available from the corresponding author [MI] upon reasonable request.
